# Structural Constraints on Human Norovirus Binding to Histo-Blood Group Antigens

**DOI:** 10.1128/mSphere.00049-16

**Published:** 2016-03-30

**Authors:** Bishal K. Singh, Mila M. Leuthold, Grant S. Hansman

**Affiliations:** Schaller Research Group at the University of Heidelberg and the DKFZ, Heidelberg, Germany; Department of Infectious Diseases, Virology, University of Heidelberg, Heidelberg, Germany; University of Pittsburgh School of Medicine

**Keywords:** X-ray crystallography, norovirus, virus structure

## Abstract

Human norovirus interacts with the polymorphic human histo-blood group antigens (HBGAs), and this interaction is thought to be important for infection. The genogroup II genotype 4 (GII.4) noroviruses are the dominant cluster, evolve every other year, and are thought to modify their binding interactions with different HBGA types. Most human noroviruses bind HBGAs, while some strains were found to have minimal or no HBGA interactions. Here, we explain some possible structural constraints for several noroviruses that were found to bind poorly to HBGAs by using X-ray crystallography. We showed that one aspartic acid was flexible or positioned away from the fucose moiety of the HBGAs and this likely hindered binding, although other fucose-interacting residues were perfectly oriented. Interestingly, a neighboring loop also appeared to influence the loop hosting the aspartic acid. These new findings might explain why some human noroviruses bound HBGAs poorly, although further studies are required.

## Opinion/Hypothesis

Human noroviruses are a major cause of acute gastroenteritis. Human noroviruses are classified into three genogroups (GI, GII, and GIV), which are further subdivided into numerous genotypes (1). The norovirus genome contains three open reading frames (ORF1 to 3), where ORF1 encodes the nonstructural proteins, ORF2 encodes the capsid protein (VP1), and ORF3 encodes a minor structural protein (VP2). Human noroviruses grow poorly in cell culture, but expression of VP1 in insect cells results in the formation of virus-like particles (VLPs) that are morphologically similar to native virions. The X-ray crystal structure of the prototype norovirus (Norwalk virus, GI genotype 1 [GI.1]) VLPs identified two domains, the shell (S) and protruding (P) domains (2). The S domain forms a scaffold surrounding the viral RNA, whereas the P domain contains the determinants of strain diversity and recognition of attachment factors. The P domain can be further subdivided into subdomains P1 and P2, and each subdomain likely has unique functions.

There is considerable antigenic drift among human noroviruses, and new genetic variants evolve every other year, which can result in new epidemics worldwide (reviewed in reference 3). It is well known that human noroviruses bind with various specificities to histo-blood group antigens (HBGAs) (4–8), which are present in saliva, on the surface of epithelial cells, and in mucosal secretions. The specific functions of HBGAs in a norovirus infection are still unclear; however, it is thought that HBGAs may act as cofactors for virus infections. At least nine different types of HBGAs are known to bind to human noroviruses, which are broadly classified into two main types, A/B/H and Lewis. Most norovirus-HBGA binding interactions can be analyzed using synthetic HBGAs, porcine gastric mucin (PGM), or saliva samples for enzyme-linked immunosorbent assay (ELISA), nuclear magnetic resonance analysis, and X-ray crystallography.

Interestingly, a number of studies have found that some human norovirus strains do not bind HBGAs. An earlier study by Huang et al. found that a GII.1 strain, termed Hawaii virus (HV), did not bind to HBGAs found in saliva and only weakly bound synthetic HBGA A and B types (8). Moreover, Harrington et al. observed that HV did not bind HBGAs in saliva or synthetic HBGAs, except for synthetic H type 1, which bound in a dose-dependent manner (4). Similarly, a GII.2 strain, termed Snow Mountain virus (SMV), also failed to bind synthetic HBGAs but weakly bound HBGA B type in saliva (4). On the other hand, SMV VLPs mixed with a 1% SMV-positive stool specimen were found to bind HBGAs and it was suggested that components in the stool specimen could support the binding to HBGAs (9). In contrast, SMV infections of human volunteers were shown to be independent of the blood type (10). At present, the GII.4 noroviruses are the dominant circulating strains and these viruses are known to bind numerous HBGA types (11–13). Conversely, the rarely detected GII.10 norovirus also binds most HBGA types, suggesting that the prevalence of strains is independent of HBGA binding (14).

**FIG 1:**
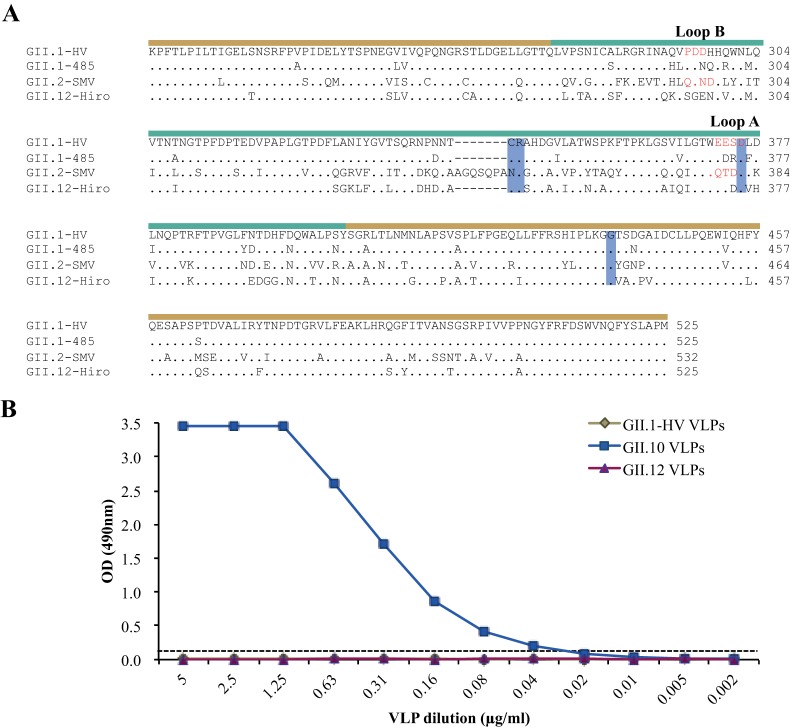
Amino acid sequence alignment and PGM ELISA study. (A) The P domain amino acid sequences of HV (GenBank accession number U07611), noro485 (DQ093065), SMV (AY134748), and GII.12 (AB044366) were aligned by Clustal X. The P1 subdomain (sand line) and the P2 subdomain (teal line) are moderately conserved. The GII.12 P domain residues interacting with the fucose moiety of HBGAs are highlighted (blue shading). Disordered amino acids in the P domain structures are red. (B) PGM ELISA of HV GII.1, GII.10, and GII.12 VLPs showed that only GII.10 VLPs bind HBGAs present in PGM. OD, optical density.

HV was the first GII.1 virus detected in 1977 (15), noro485 (GII.1) was discovered in ~2005 (16), and SMV (GII.2) was identified in 1982 (17). At present, the GII.1 and GII.2 noroviruses are only minor causes of gastroenteritis in humans, although previously these viruses caused a significant number of outbreaks. The amino acid sequence identity of GII.1 (HV and 485) and GII.2 (SMV) is approximately 74%. Phylogenetic analysis of VP1 amino acid sequences has indicated that GII.1 noroviruses are closely related to GII.12 noroviruses (16), having an amino acid identity of 85% (Fig. 1A and data not shown).

To confirm previously determined HBGA interactions, we expressed VP1 of HV GII.1 (GenBank accession number U07611), GII.10 (AF504671), and GII.12 (AB044366) in insect cells and examined VLP binding to PGM by ELISA (18). VLPs were expressed and purified as previously described (16). Microtiter plates (MaxiSorp; Thermo Scientific) were coated with 100 µl/well PGM (10 µg/ml; Sigma) for 4 h at room temperature. Plates were washed three times with phosphate-buffered saline (PBS; pH 7.4) containing 0.1% Tween 20 (PBS-T) and blocked with 5% skim milk in PBS (PBS-SM) overnight at 4°C. Plates were washed three times with PBS-T, VLPs at 5 µg/ml were 2-fold serially diluted, and then 100 µl of each dilution was added to triplicate wells for 2.5 h at 18°C. After washing with PBS-T, primary polyclonal antibodies (HV-1068 [specific for GII.1] and 026-Rab2 [binds both GII.10 and GII.12]) at 100 µl/well were added at dilutions of 1:6,400 and 1:12,800 in PBS-T-SM, respectively. After incubation for 1.5 h at 18°C, a horseradish peroxidase-conjugated anti-rabbit secondary antibody (Thermo Scientific) was added at a 1:40,000 dilution in PBS-T-SM and the mixture was incubated overnight at 4°C. Plates were processed as previously described (19). The cutoff was set at an optical density at 490 nm of >0.15, which was about three times the value of the negative control (PBS). The ELISA data showed that GII.10 VLPs at 0.04 µg/ml were able to bind to PGM, whereas GII.1 and GII.12 VLPs did not bind to PGM at any concentration (Fig. 1B). This result corresponded well to previous findings, except that the GII.12 P domain was shown to bind the B-type HBGA (B trisaccharide) by X-ray crystallography (Protein Data Bank ID 3R6K) (14). Closer inspection of the GII.12 P domain B trisaccharide complex structure revealed that the electron density of the B trisaccharide was weaker than that of other norovirus B trisaccharide complex structures at similar resolutions (13, 14, 20), suggesting that the GII.12 P domain only weakly bound the B trisaccharide. Moreover, according to previous characterization of PGM, the abundance of the B trisaccharide in PGM (21) might not be sufficient to be recognized by GII.12 VLPs.

To elucidate the constraints on non-HBGA binders from a structural perspective, we determined the X-ray crystal structures of the capsid-protruding domains (P domains) of two GII.1 (HV and noro485) nonbinders and one GII.2 (SMV) HBGA nonbinder. The P domains of HV, noro485 (DQ093065), and SMV (AY134748) were expressed in *Escherichia coli* and purified as previously described (14). Crystals were grown in hanging drops with a 1:1 mixture of protein sample (~4 mg/ml) and mother solution for 2 to 6 days at 18°C. P domains of HV crystallized in 20% (wt/vol) PEG 3350 and 0.2 M KCl, noro485 crystallized in 20% (wt/vol) PEG 3350 and 0.2 M magnesium formate, and SMV crystallized in 20% PEG 2000 and 0.1 M Tris-HCl (pH 7.0). Prior to flash freezing, crystals were transferred to a cryoprotectant containing 30% ethylene glycol in mother solution. We also attempted to cocrystallize these P domains with various concentrations of HBGAs (Dextra, United Kingdom) under different mother liquor conditions; however, we did not observe any electron density for the HBGAs (data not shown). Data collection, structure solution, and refinement of the unbound structures were performed as previously described (13). Statistics of data collection and refinement are shown in Table 1.

**FIG 2:**
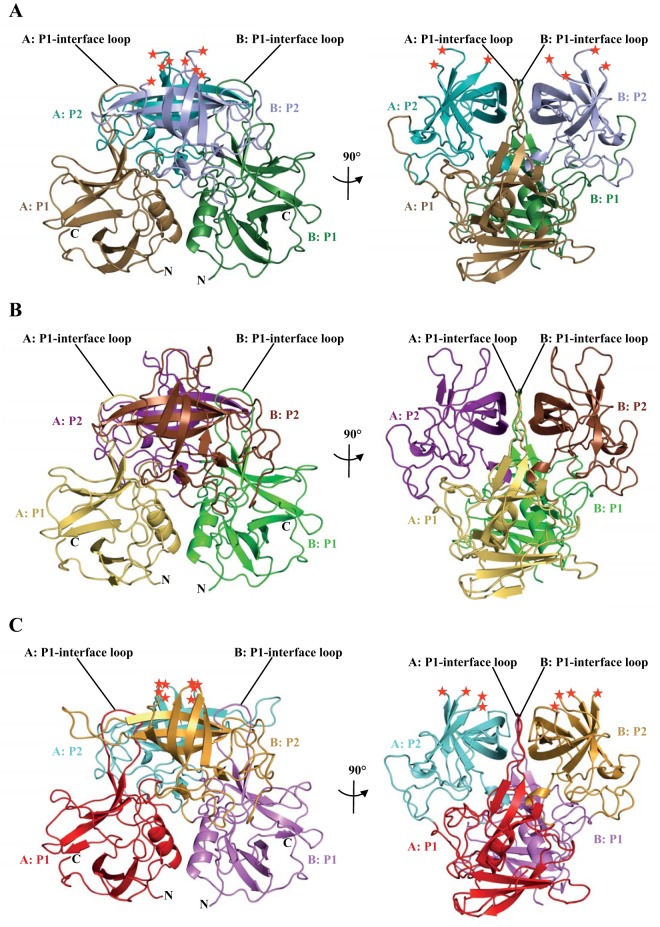
X-ray crystal structures of HV, noro485, and SMV P domains. Cartoon representations of the HV, noro485, and SMV P domains are shown. The red asterisks represent the loops missing because of a lack of electron density (cutoff at 3σ). (A) HV GII.1 is colored according to monomers (chains A and B) and P1 and P2 subdomains, i.e., chain A (P1 subdomain, sand; P2 subdomain, teal) and chain B (P1 subdomain, forest green; P2 subdomain, light blue). (B) noro485 GII.1 chain A (P1 subdomain, yellow orange; P2 subdomain, purple) and chain B (P1 subdomain, green; P2 subdomain, brown). (C) SMV GII.2 chain A (P1 subdomain, red; P2 subdomain, aquamarine) and chain B (P1 subdomain, violet; P2 subdomain, bright orange).

HV, noro485, and SMV P domains diffracted to 1.86, 1.50, and 1.60 Å resolutions, respectively (Table 1). Similar to the P1 subdomains of other human noroviruses, those of HV, noro485, and SMV comprised β-sheets and one α-helix, while the P2 subdomains contained six antiparallel β-strands that formed a barrel-like structure (Fig. 2). Superposition of these three P domains revealed similar overall structures with a root mean square deviation (RMSD) between 0.56 and 0.97 Å (data not shown). The GII.12 P domain was also structurally similar to HV, noro485, and SMV structures, with RMSDs of ~0.57 (HV and noro485) and 0.82 Å (SMV) (14). Compared to the P domains of HV and noro485, that of SMV contained an insertion loop (SMV numbering; Ala345 to Ala351) in the P2 subdomain (Fig. 1A), which was also observed in a GII.10 P domain structure (14).

**TABLE 1:** GII.1 and GII.2 P domain data collection and refinement statistics

Statistics	HV GII.1 (4ROX)	485 GII.1 (4RPD)	SMV GII.2 (4RPB)
Data collection statistics^a^			
Space group	C_2_	C_2_	C_2_
Cell dimensions			
*a*, *b*, *c* (Å)	139.06, 78.16, 74.66	99.41, 73.50, 81.81	166.64, 96.17, 64.12
α, β, γ (°)	90, 122.42, 90	90, 94.11, 90	90, 90.01, 90
Resolution range (Å)	37.33–1.86 (1.93–1.86)^b^	48.84–1.50 (1.55–1.50)	41.66–1.60 (1.66–1.60)
*R*_merge_ (%)	6.15 (66.89)	6.53 (61.73)	6.39 (74.52)
Avg *I*/σ*I*	13.25 (2.00)	14.23 (2.06)	15.13 (2.05)
Completeness (%)	99.20 (98.89)	98.80 (96.29)	98.90 (95.36)
Redundancy (fold)	3.7 (3.7)	4.5 (4.2)	3.7 (3.7)
Refinement statistics			
Resolution range (Å)	37.33–1.89	48.84–1.51	41.66–1.61
No. of reflections	53,930	91,190	129,667
*R*_work_/*R*_free_	17.73/20.13	14.64/18.67	16.50/18.16
No. of atoms	4,936	9,750	14,177
Protein	4,602	9,098	13,459
Ligand/ion	28	0	28
Water	306	652	690
Avg *B* factors (Å^2^)			
Protein	37.50	20.60	30.80
Ligand/ion	43.60	0	40.40
Water	40.60	29.20	34.30
RMSD			
Bond length (Å)	0.006	0.009	0.006
Bond angle (°)	1.01	1.25	1.06

^a^ Data sets were collected from single crystals.

^b^ Values in parentheses are for the highest-resolution shell.

**FIG 3:**
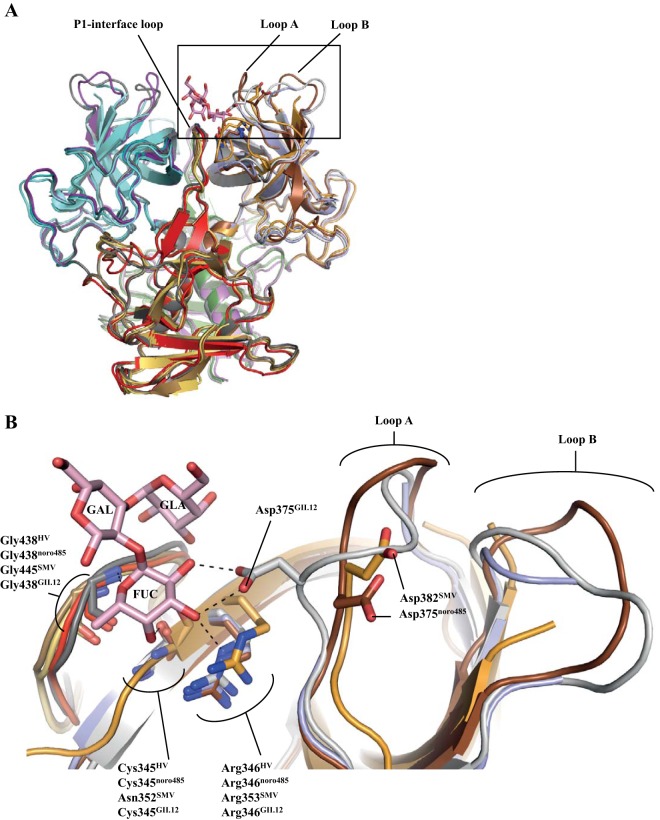
Structural constraints on HV, SMV, and noro485 binding to HBGAs. (A) Cartoon representation and superposition of the HV, noro485, SMV, and GII.12 P domains, which are colored as in Fig. 2, while the GII.12 P domain is dark gray (chain A) or light gray (chain B). (B) Close-up of the HBGA pocket (boxed region) showing the GII.12 P domain residues (gray) that interact with the fucose moiety (FUC; light pink sticks). The equivalent residues of HV, noro485, and SMV are similarly oriented, except for Asp375 of noro485 and Asp382 of SMV, which point ~180° away from the fucose moiety. The loops hosting Asp (Asp375 of HV, Asp375 of noro485, Asp382 of SMV, and Asp375 of GII.12) and the neighboring loops are labeled A and B, respectively. Hydrogen bonds are shown as black dashed lines.

The high resolution of these structures allowed us to clearly define protein side chains and water molecules. In the HV and SMV P domains, we found two loop regions with disordered residues in the P2 subdomain, i.e., no discernible electron density, which suggested a certain degree of flexibility in these loops. In the HV P domain, Pro295 to Asp296 and Glu372 to Asp375 were disordered, while in the SMV P domain, residues Gln295 to Asp298 and Trp378 to Asp381 were disordered (Fig. 2). In several GII noroviruses, a common set of residues usually binds the fucose moiety of HBGAs through direct hydrogen bonds (13, 14). In the GII.10 P domain, these are Asn355 (main chain), Arg356 (side chain), Asp385 (side chain), and Gly451 (main chain) (14). In the GII.12 P domain, Cys345 (main chain), Arg346 (side chain), Asp375 (side chain), and Gly438 (main chain) primarily stabilized the fucose moiety. The equivalent residues in the HV P domain were Cys345, Arg346, Asp375, and Gly438; in noro485, they were Cys345, Arg346, Asp375, and Gly438; and in SMV, they were Asn352, Arg353, Asp382, and Gly445 (Fig. 3). Moreover, the GII.1 and GII.2 P domain structures all contained the P1 interface loop that was similar in size and orientation to the GII.10 and GII.12 structures, and this was shown to be important for HBGA binding (Fig. 2 and 3). The high conservation in the common set of residues that bind fucose and the presence of a P1 interface loop similar to that of HBGA binders suggested that GII.1 and GII.2 were potentially capable of binding HBGAs.

Superposition of the HV, noro485, SMV, and GII.12 P domains revealed that the main and side chains of this common set of residues mostly overlapped the equivalent GII.12 residues, except for Asp (Asp375 in HV, Asp375 in noro485, and Asp382 in SMV). In the noro485 and SMV structures, Asp375 and Asp382, respectively, were pointing ~180° away from the fucose, whereas in the HV structure, Asp375 was missing because of the lack of electron density. Additionally, in the noro485 P domain structure, the polypeptide backbone of the loop hosting Asp375 (termed loop A) superimposed poorly onto the corresponding GII.12 P domain (Fig. 3). This altered orientation of loop A appeared to be partially adopted by SMV, although the precise orientation was difficult to determine because of the lack of electron density. Moreover, a neighboring loop (termed loop B) may have influenced the orientation and stability of loop A. For example, in noro485, loop B was oriented differently from that of GII.12. In HV and SMV, loop B could not be modeled into the structures because of the lack of electron density. These data suggest that loop B might influence the stability of loop A, since loop B was missing from HV and partially missing from SMV. These observations suggest that fine-tuning of loop B may also regulate the orientation of loop A, which in turn influences the orientation of the Asp, although direct evidence is lacking.

It is well known that substitutions of the common set of residues interacting with the fucose moiety of the HBGAs can greatly reduce binding (22, 23). However, our new data indicate that Asp375 in HV, Asp375 in noro485, and Asp382 in SMV are unable to participate in the stabilization of the fucose moiety of HBGAs because of their flexibility or suboptimal orientation. The reason(s) for norovirus binding or not binding to HBGAs remains unclear, although a recent report showed that HBGAs enhance GII.4 norovirus infections in cell culture (24). Perhaps the HBGA interactions are important for some norovirus strains, while other strains do not interact with HBGAs at all.
